# Comorbidity and adverse events in acquired hemophilia A: data from the GTH-AHA-EMI study

**DOI:** 10.1016/j.rpth.2024.102565

**Published:** 2024-09-05

**Authors:** Christian Herbert Burgmann, Ulrich J. Sachs, Karolin Trautmann-Grill, Christian Pfrepper, Paul Knöbl, Richard Greil, Johannes Oldenburg, Wolfgang Miesbach, Katharina Holstein, Hermann Eichler, Patrick Möhnle, Matthias Höpting, Christiane Dobbelstein, Robert Klamroth, Andreas Tiede

**Affiliations:** 1 Hematology, Hemostasis, Oncology, and Stem Cell Transplantation, Hannover Medical School, Hannover, Germany; 2 Institute for Clinical Immunology and Transfusion Medicine, Justus Liebig University, Giessen, Germany; 3 Medical Clinic I, University Hospital Carl Gustav Carus, Technical University Dresden, Dresden, Germany; 4 Division of Hemostaseology, Medical Department I, University Hospital Leipzig, Leipzig, Germany; 5 Department of Medicine 1, Division of Hematology and Hemostasis, Medical University of Vienna, Vienna, Austria; 6 Medical Department III, Paracelsus Medical University Salzburg, Salzburg Cancer Research Institute-Center for Clinical Cancer and Immunology Trials, Cancer Cluster Salzburg, Salzburg, Austria; 7 Institute of Experimental Hematology and Transfusion Medicine, University Clinic Bonn, Bonn, Germany; 8 Medical Clinic II, Institute of Transfusion Medicine, Goethe University, Frankfurt, Germany; 9 Hematology and Oncology, University Medical Center Hamburg-Eppendorf, Hamburg, Germany; 10 Institute of Clinical Hemostaseology and Transfusion Medicine, Saarland University and University Hospital, Homburg/Saar, Germany; 11 Department of Transfusion Medicine, Cellular Therapeutics and Hemostaseology, Hospital of Ludwig Maximilian University, Munich, Germany; 12 Department of Anesthesiology, Hospital of Ludwig Maximilian University, Munich, Germany; 13 Department of Hematology and Oncology, University Hospital Regensburg, Regensburg, Germany; 14 Internal Medicine, Vivantes Clinic Friedrichshain, Berlin, Germany

**Keywords:** antibodies, bispecific, drug-related side effects and adverse reactions, factor 8 deficiency, acquired, frailty, hemorrhage

## Abstract

**Background:**

Persons with acquired hemophilia A are often older and suffer from comorbidity or frailty. Little is known about the impact on clinically relevant outcomes of acquired hemophilia A.

**Objectives:**

To assess the relevance of age, physical performance status, comorbidity, and concomitant medication on the risk of bleeding and other outcomes.

**Methods:**

Post hoc analysis of data from the GTH-AHA-EMI study that used emicizumab for bleed protection and withheld immunosuppressive treatment during the early phase of management. Primary endpoint was the rate of clinically relevant new bleeding (CRNB) during the first 12 weeks of emicizumab prophylaxis.

**Results:**

Forty-seven patients were enrolled. Median age was 76 years; performance status (World Health Organization performance status [WHO-PS]) was 3 or worse in 41%; Charlson comorbidity index (CCI) was 5 or higher in 63%; antithrombotic drugs were reported in 34%. Rate of CRNB during 12 weeks of emicizumab prophylaxis was similar across subgroups of age, sex, WHO-PS, CCI, baseline factor VIII activity, and inhibitor titer. Patients with CRNB during the study had more severe anemia already at baseline. However, persistent severe anemia in week 4 was not related to risk of bleeding beyond this time. CRNB was associated with injury from falling in 7 of 14 patients. Adverse events grade 3 or higher were not related to baseline CCI or age but were more frequent in patients with poor WHO-PS.

**Conclusion:**

Emicizumab provided bleed protection regardless of age and comorbidity. Clinical baseline characteristics did not predict breakthrough bleeding under emicizumab. Poor WHO-PS at baseline was associated with severe adverse events during the study.

## Essentials

• Persons with acquired hemophilia are often frail and suffer from comorbidity.
• Emicizumab is increasingly used to prevent bleeding in acquired hemophilia A in these patients.
• Protection from bleeding was similar across subgroups of age, sex, and comorbidity.
• The risk of nonbleeding adverse events was higher in patients with poor performance status.

## 1 Introduction

Acquired hemophilia A (AHA) is a serious bleeding disorder caused by neutralizing autoantibodies against coagulation factor (F)VIII [1,2]. AHA is a rare disorder with an estimated incidence between 1.5 and 6 cases per million per year [3,4]. The age distribution is bimodal, showing a small peak in women around the age of 30 to 40 years, where AHA is usually related to the postpartum period, and a larger peak in men and women of 70 to 90 years [5]. In most registries, the median age at presentation is approximately 75 years of age [3,[5], [6], [7], [8], [9], [10], [11], [12]], with the exception of a study from China, where patients had a mean age of 52 years [13]. Thirty percent to 50% of patients have concomitant autoimmune or malignant disorders, usually considered to be underlying conditions linked to the occurrence of AHA [5].

The traditional management approach of AHA includes (I) treatment of acute bleeds with bypassing agents (recombinant FVIIa and activated prothrombin complex concentrate) or recombinant porcine FVIII (susoctocog alfa) and (II) immunosuppressive therapy (IST) to eradicate autoantibody formation and to induce remission of the disease [14]. While bleed treatment with bypassing agents and susoctocog alfa is usually effective and safe, registries showed that IST was often related to infections and other adverse events [[6], [7], [8],^15^]. In fact, infections have consistently been reported as the leading cause of death in Western European persons with AHA. Attempts to reduce complications by less intense IST have not always been successful [16].

While advanced age, frailty, and comorbidity have often been discussed as risk factors for adverse events of IST, evidence for this hypothesis is limited. The GTH-AH 01/2010 observational study, which used IST in all patients immediately after diagnosis, found that mortality was related to low FVIII activity, underlying malignancy, and poor physical performance status at baseline [6]. Age per se was not a risk factor. Low FVIII activity at baseline was also related to a much longer time needed to achieve remission, higher overall exposure to IST, and recurrent bleeding, which may explain why it was related to mortality, although fatal bleeding was actually rare. The overall conclusion of this and other observation studies was that intense and prolonged IST is the main risk for persons with AHA.

Recently, the bispecific monoclonal antibody emicizumab has been introduced into the management of AHA [17,18]. This drug can replace the function of FVIIIa, can be given subcutaneously, and is suitable for outpatient management [19,20]. Several case reports [[21], [22], [23]], observational studies [24,25], and 2 clinical trials [26,27] reported that emicizumab prevented bleeds in most persons with AHA. The GTH-AHA-EMI study performed by our group used emicizumab for the early phase of AHA management without concomitant IST [27]. Low rates of breakthrough bleeding were reported, but promising data with regard to the risk of severe or fatal infection and overall survival were also reported [28]. It was hypothesized that postponing IST during the initial phase of management contributed to these favorable outcomes. The downside of this improvement was that very few patients achieved remission of AHA spontaneously and a small but persistent risk of breakthrough bleeding was noted during the entire study period. This included 2 occurrences of fatal bleeding, both occurring after discharge from the hospital.

Therefore, it would be helpful to identify patients at risk of breakthrough bleeding or other adverse events and to consider suitable protective measures. To this end, the current post hoc analysis of data from the GTH-AHA-EMI clinical trial was done. Our objective was to analyze not only clinical characteristics such as age, comorbidity, and physical performance status but also laboratory parameters and concomitant medication as potential risk factors for bleeding and other clinically relevant adverse events in persons with AHA under prophylaxis with emicizumab.

## 2 Methods

### 2.1 Study oversight

This study included patients from the GTH-AHA-EMI phase 2 multicenter clinical trial (NCT04188639, registered at www.clinicaltrials.gov). The objectives, eligibility criteria, and endpoints of this study have been published [27]. Persons with AHA were treated with emicizumab according to the study protocol with a loading dose of 6 mg/kg of body weight (day 1) and 3 mg/kg (day 2) subcutaneously, followed by a maintenance dose of 1.5 mg/kg per week from day 8 onwards until week 12. IST was withheld during the first 12 weeks after starting emicizumab. Thereafter, it was at the discretion of the investigator. All patients gave written informed consent before enrollment. All study procedures were in accordance with the International Conference on Harmonization Guidelines for Good Clinical Practice and were approved by the regulatory authorities in Germany and Austria and by the ethics committees of all participating centers before initiation.

### 2.2 Definition of endpoints

The primary endpoint was the number of clinically relevant new bleeding (CRNB) per patient-week after the first dose of emicizumab until week 12 after starting emicizumab treatment or dropout, whatever occurred first. A bleed was defined as clinically relevant if it required intervention by a healthcare professional or if it caused pain or any other kind of disturbance in the patient’s daily life. A bleed was considered new if it occurred either for the first time in an anatomical region or ≥72 hours after the last treatment of a previous bleed in the same region. If 2 or more bleeds occurred simultaneously in different anatomical regions, they were counted as separate events. CRNB were further classified according to severity (clinically relevant nonmajor or major, according to International Society on Thrombosis and Haemostasis criteria), cause (spontaneous, traumatic, or related to surgery), and treatment (untreated or treated with factor concentrate). Secondary endpoints were adverse events of the Medical Dictionary for Regulatory Activities system organ classes and mortality within 24 weeks after enrollment.

### 2.3 Indices of comorbidity and physical performance

The 19-item version of the Charlson comorbidity index (CCI) was used [29]. A score of 1 point each was assigned for myocardial infarction, chronic heart failure, peripheral vascular disease, cerebrovascular disease, dementia, chronic pulmonary disease, connective tissue disease, ulcer disease, mild liver disease, or uncomplicated diabetes mellitus; 2 points for hemiplegia, moderate or severe renal disease, diabetes with end organ damage, any tumor without metastasis, leukemia, or lymphoma; 3 points for moderate or severe liver disease; and 6 points for metastatic solid tumor or AIDS. Presence or absence of relevant diseases was assessed from systematic review of all medical history items collected at baseline. The points were summed up, and one point was added for every decade over the age of 50 years (maximum 4 points), resulting in the total score.

The World Health Organization performance status (WHO-PS) was assigned at baseline by local investigators according to standard definitions: 0, asymptomatic (fully active, able to carry on all predisease activities without restriction); 1, symptomatic but completely ambulatory (restricted in physically strenuous activity but ambulatory and able to carry out work of a light or sedentary nature; eg, light housework, office work); 2, symptomatic, <50% of time spent in bed during the day (ambulatory and capable of all self-care but unable to carry out any work activities; up and about more than 50% of waking hours); 3, symptomatic, >50% of time spent in bed, but not bedbound (capable of only limited self-care, confined to bed or chair 50% or more of waking hours); 4, bedbound (completely disabled, cannot carry on any self-care, totally confined to bed or chair); 5, death. Hospitalization because of newly diagnosed AHA or study enrollment did not per se define a score of 2 or higher; instead, the actual physical condition of the patient was considered for scoring.

### 2.4 Laboratory data

The laboratory tests were done by study sites at baseline (visit [V]1), week 4 (V5), and week 13 (V7) and included the following: hematology (red blood cells [RBCs], red blood indices [mean corpuscular hemoglobin {MCH}, mean corpuscular volume, and mean corpuscular hemoglobin concentration], hemoglobin [Hb], hematocrit, leukocytes, neutrophils, lymphocytes, monocytes, eosinophils and basophils, platelets, and absolute neutrophil count), coagulation (FVIII activity [chromogenic assay with bovine components] and FVIII inhibitor [only V1]), and clinical chemistry (urea, creatinine, aspartate aminotransferase, alanine aminotransferase, alkaline phosphatase, gamma-glutamyltransferase, bilirubin, calcium, potassium, sodium, magnesium, inorganic phosphate, chloride, glucose, lactate dehydrogenase, C-reactive protein, and glucose). Data were retrieved from the study database.

### 2.5 Statistical analysis

Data were aggregated and reported using medians, IQRs, ranges, numbers, and proportions as appropriate. Fisher’s exact test, Pearson’s chi-squared test, or Wilcoxon test were used to compare proportions and continuous data as appropriate. A *P* value of <.05 was considered for statistical significance. The mean rate of CRNB per patient-week and its 95% CI were estimated using generalized linear models with negative binomial distribution. R version 4.3.0 (R Foundation for Statistical Computing) and tidyverse (2.0.0) (Hadley Wickham; https://tidyverse.tidyverse.org/) were used for analysis, with the MASS package (7.3-60.0.1) for generalized linear models.

## 3 Results

### 3.1 Patient population and baseline characteristics

All 47 patients enrolled in the GTH-AHA-EMI were included in this analysis. Age, sex distribution, and underlying disorders were similar to previous Western European cohorts of persons with AHA (Table). Body mass index was normal (18.5-24.9 kg/m^2^; *n* = 19) or overweight (25-29.9 kg/m^2^; *n* = 16) in most patients, while few patients were underweight (<18.5 kg/m^2^; *n* = 5) or obese (≥30 kg/m^2^; *n* = 7). WHO-PS indicated good physical performance in the minority of patients (WHO-PS, 0-1; *n* = 15), while most were slightly (WHO-PS, 2; *n* = 13) or more severely compromised (WHO-PS, 3-4; *n* = 19). CCI was low in very few patients (CCI, ≤2; *n* = 6) but moderately increased (CCI, 3-4; *n* = 11) or severely increased (CCI, ≥5; *n* = 30) in the majority of patients. Before starting emicizumab prophylaxis, all 47 patients had 1 or more recorded bleeding events, and 37 (79%) had required treatment with bypassing agents or susoctocog alfa.

**Table:** Selected baseline characteristics of the study population.

Characteristic	Values (*N* = 47)
Age (y)	
Median (IQR)	76 (66-80)
Range	21-93
>75 y	24 (51%)
≤75 y	23 (49%)
Sex	
Female	23 (49%)
Male	24 (51%)
Race	
Caucasian	46 (98%)
Not reported	1 (2%)
Underlying disorder	
None (idiopathic)	33 (70%)
Autoimmunity	7 (15%)
Malignancy	6 (13%)
Postpartum period	1 (2%)
Body mass index (kg/m^2^)	
Median (IQR)	25 (22-29)
Range	16-38
<18.5 kg/m^2^	5 (11%)
18.5-24.9 kg/m^2^	19 (40%)
25-29.9 kg/m^2^	16 (34%)
≥30 kg/m^2^	7 (15%)
WHO performance status	
Median (IQR)	2 (1-3)
Range	0-4
WHO performance status 0	4 (8.5%)
WHO performance status 1	11 (23%)
WHO performance status 2	13 (28%)
WHO performance status 3	12 (26%)
WHO performance status 4	7 (15%)
CCI	
Median (IQR)	5 (4-7)
Range	0-13
CCI 0-2	6 (13%)
CCI 3-4	11 (23%)
CCI 5-7	19 (40%)
CCI 8-13	11 (23%)

Data are presented as *n* (%) unless stated otherwise.

CCI, Charlson comorbidity index; WHO, World Health Organization.

### 3.2 Comorbidity

**Figure 1:**
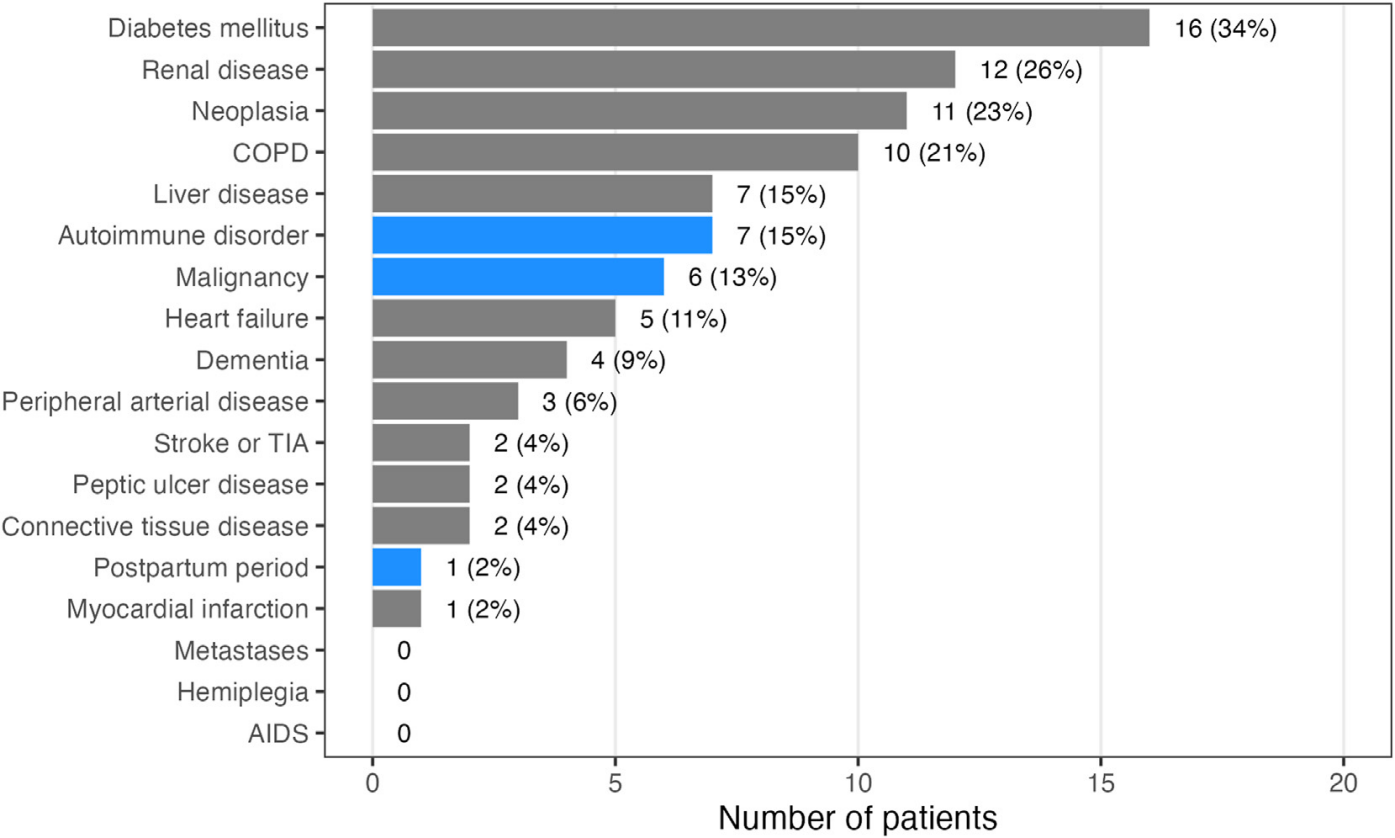
Baseline concomitant disorders and acquired hemophilia A (AHA)–associated disorders. Bars show numbers of patients with percent (out of 47 enrolled patients) denoted next to each bar. Gray fills indicate concomitant disorders that are part of the Charlson comorbidity index. Blue fills indicated additional AHA-related disorders. Note that “neoplasia” (gray bar) contains all concomitant neoplastic disorders, while “malignancy” (blue bar) denotes only those malignancies that were considered associated with AHA. COPD, chronic obstructive pulmonary disorder; TIA, transient ischemic attack.

The most frequent comorbidities at baseline were diabetes mellitus, renal disorders, neoplasia, and chronic obstructive pulmonary disorder (Figure 1). Heart failure, peripheral arterial disease, previous stroke/transitory ischemia attack, and myocardial infarction were less often reported. Underlying disorders of AHA were separately recorded and did not completely overlap with the concomitant disorders used for CCI. For instance, among the 6 AHA-associated malignancies, there were 2 cases of monoclonal gammopathy that were not recorded as neoplasia in the medical history. Conversely, not all neoplastic disorders were considered AHA-associated malignancies, eg, cases of basal cell carcinoma, uterine fibrotid, or colorectal carcinoma in long-standing complete remission.

**Figure 2:**
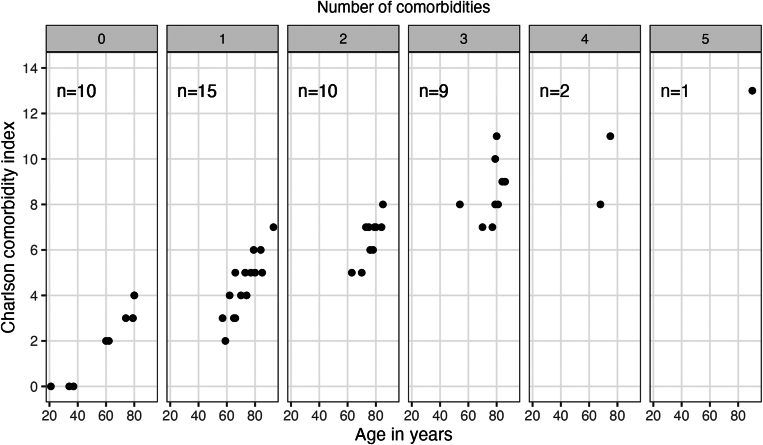
Charlson comorbidity index according to age and number of concomitant disorders. Patients (represented by 1 point each) are grouped into facets according to number of comorbidities (0-5), and their Charlson comorbidity index is shown according to age.

CCI by definition increased with age and number of comorbidities (Figure 2). Ten (21%) of the 47 patients had no comorbidities (CCI, 0-4; depending on age). This group included, for instance, a 37-year-old patient with pregnancy-associated AHA (CCI, 0). On the other end of the spectrum, there were 12 (26%) patients with ≥3 comorbidities (CCI, ≥7), including a 90-year-old patient with dementia, heart failure, and renal, pulmonary, and liver disorders (CCI, 13).

Atrial fibrillation or flutter is not part of the CCI and was reported in 4 (9%) of the 47 patients at baseline. In addition, 1 patient had a brief period of atrial fibrillation on days 2 and 3 after starting emicizumab.

### 3.3 Antithrombotic drugs

Antithrombotic drugs were reported in 16 (34%) of the 47 patients. Acetylsalicylic acid (ASA) was reported in 11 (23%). It had been stopped around the time of study enrollment in all patients (days −18 to +1) and was later restarted in 3 of them (days 5, 26, and 52). Direct FXa inhibitors and low-molecular-weight heparin (LMWH) were reported in 3 (6%) and 7 (15%) patients, respectively. Direct FXa inhibitors were stopped before enrollment (days −10, −21, and −271) and not restarted during the study. LMWH was administered in prophylactic doses before study entry in 3 patients (stopped on days −10, −8, and −2) and was used for short-term prophylaxis after starting emicizumab in the remaining 4 patients.

### 3.4 Bleeding rate according to baseline characteristics

**Figure 3:**
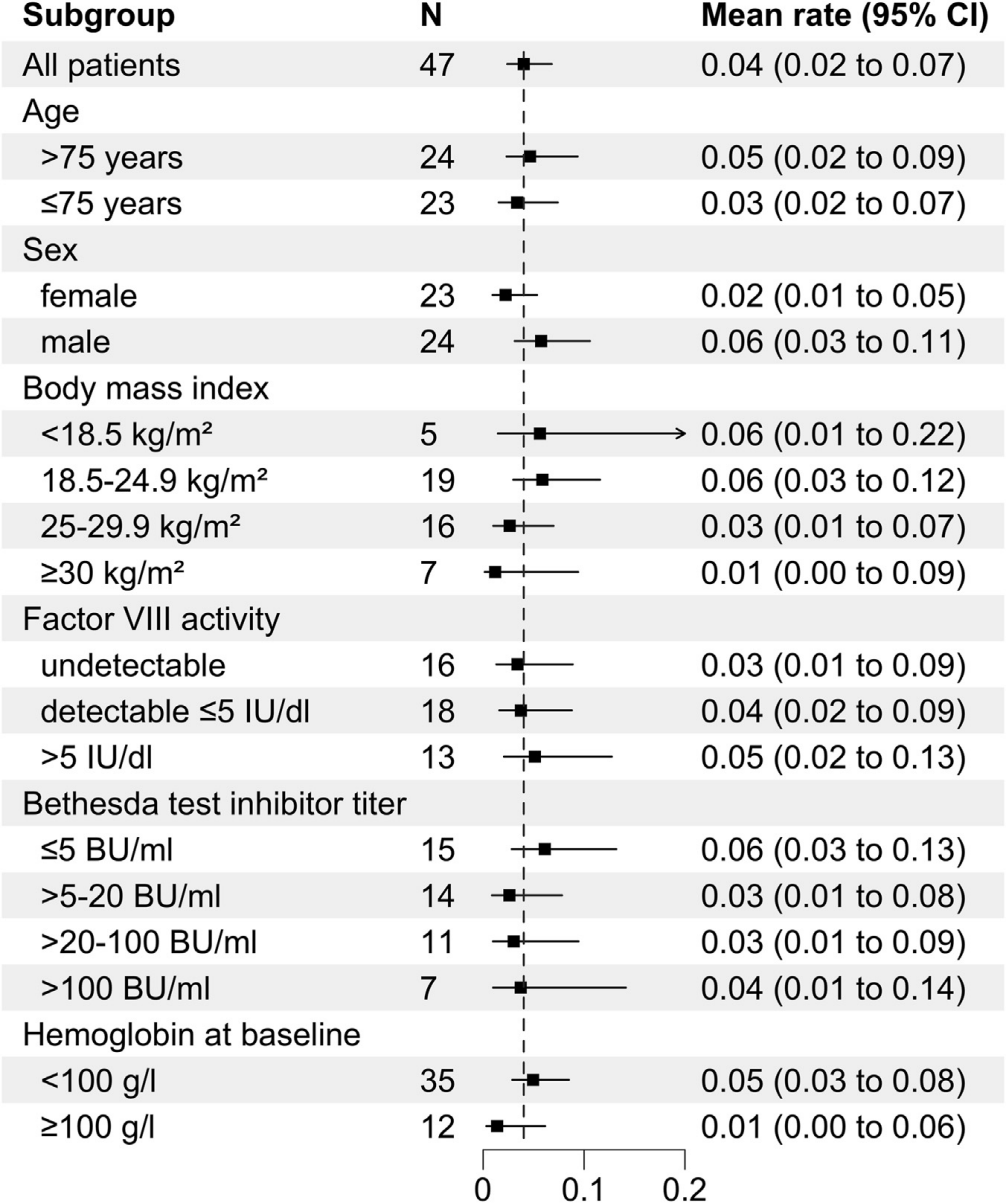
Bleeding rate in 12 weeks after starting emicizumab according to baseline demographic and laboratory characteristics. Data show mean (square symbol) and 95% CIs (whisker) according to a negative binomial distribution model. The dashed vertical line indicates the mean of the entire study population. BU, Bethesda unit.

Twenty-two CRNBs were recorded in 14 of the 47 patients between the day of starting emicizumab and the end of week 12 or dropout. The mean rate was 0.04 CRNBs per patient-week (negative binomial model; 95% CI, 0.02-0.07). Little variation was observed comparing subgroups of age, sex, baseline FVIII activity, or baseline inhibitor titer (Figure 3). Patients with higher body mass index tended to have fewer bleeds, but CIs were largely overlapping. Patients with baseline Hb ≥100 g/L tended to have a lower bleeding rate (mean 0.01 [95% confidence interval, 0.00-0.06]) compared with patients with Hb <100 g/L (mean 0.05 [95% confidence interval, 0.03-0.08]).

**Figure 4:**
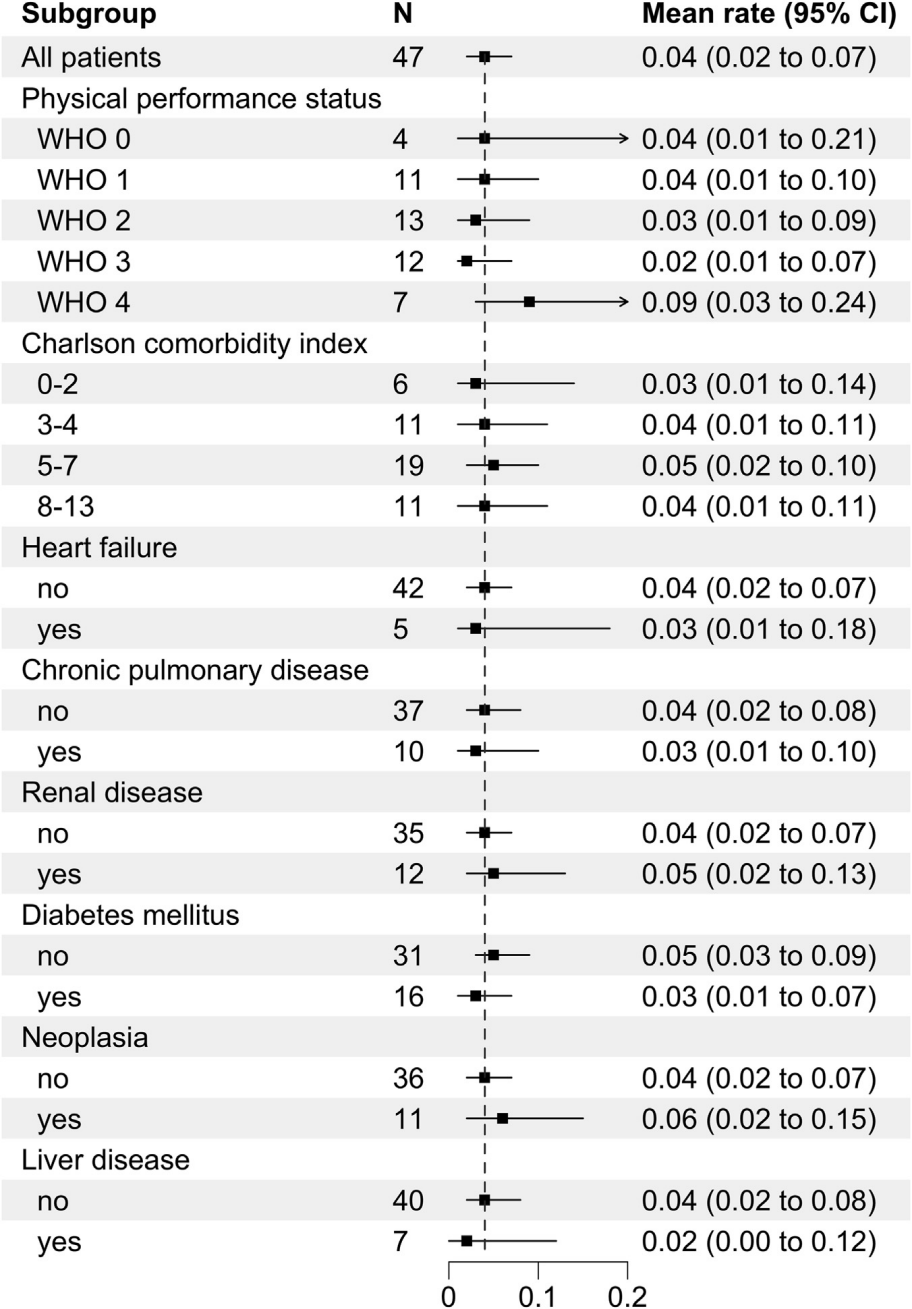
Bleeding rate in 12 weeks after starting emicizumab according to selected concomitant disorders and World Health Organization (WHO) performance status at baseline. Data show mean (square symbol) and 95% CIs (whisker) according to a negative binomial distribution model. The dashed vertical line indicates the mean of the entire study population.

No difference was observed according to WHO-PS, CCI, or any single concomitant disorder at baseline (Figure 4). Of note, both the 37-year-old patient (CCI, 0) and the 90-year-old patient (CCI, 13) mentioned above had no bleeding event during the study, illustrating that comorbidity did not increase the bleeding risk while under emicizumab.

Comparing dichotomized baseline characteristics in patients with and without bleeding during the study provided similar results (Supplementary Table S1).

### 3.5 Bleeding events related to antithrombotic drugs

The 2 patients stopping ASA on day −2 and day 1 of emicizumab prophylaxis had CRNB on days 1 and 2, respectively. One patient starting ASA on day 52 had 2 bleeds on days 52 and 57. Emicizumab had been discontinued and removed by plasmapheresis in this patient 2 weeks earlier because of mesenteric infarction. Histology later confirmed that the event had not been thromboembolic but rather resulted from cholesterol embolization. However, this was not known to the investigator at the time of treatment. The bleed on day 52 was at the site of previous surgery, and the bleed on day 57 was a gastrointestinal bleed. In retrospect, these bleeds were related to stopping and removal of emicizumab and perhaps the administration of ASA. In summary, 3 patients had bleeding associated with ASA, but all of them had presumably low or absent emicizumab levels at this time.

One of the patients receiving prophylactic dose LMWH from day 6 to 9 had a bleed on day 14. Another patient receiving prophylactic dose LMWH from day 1 to 4 and 34 to 36 had 2 bleeds on day 18. The remaining 2 patients received LMWH prophylaxis during the study without bleeding. In summary, LMWH prophylaxis was not related to bleeding while under emicizumab.

### 3.6 Anemia and CRNB

Having observed a trend toward higher rates of CRNB in patients with baseline Hb <100 g/L, we were interested in comparing the complete blood counts and other baseline laboratory parameters in patients with and without CRNB while under emicizumab. This analysis confirmed that patients with bleeding during the study had significantly lower Hb (84 vs 97 g/L; *P* = .03), RBCs, and MCH at baseline (Supplementary Table S2).

**Figure 5:**
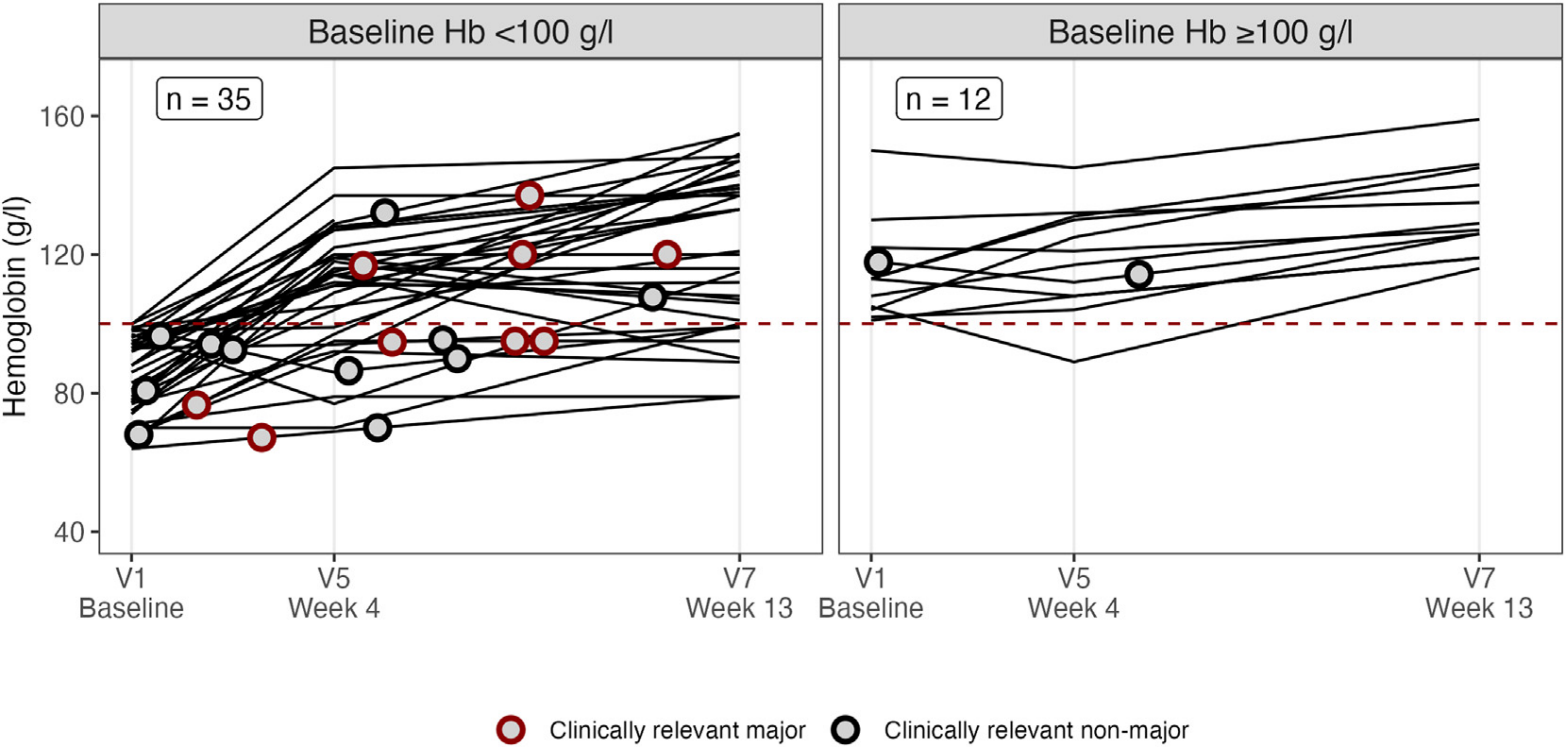
Clinically relevant new bleeding according to hemoglobin (Hb) at baseline and during the study. Lines show the course of Hb values taken from local laboratory complete blood counts at scheduled visits. Red and blue circles indicate major and nonmajor clinically relevant new bleeding, respectively. They were placed according to time of occurrence (X axis) and Hb value interpolated between adjacent visits (Y axis). Note that this interpolation was made under the assumption that the change of Hb between 2 visits was linear. V, visit.

To gain some deeper insight into the relationship between anemia and CRNB, we plotted Hb values and bleeding events over time (Figure 5). Most bleeding events seemed to have occurred while the Hb was already rising or corrected. Specifically, 24 of the 35 patients with baseline Hb <100 g/L had improved to ≥100 g/L by week 4, but still 5 (21%) of them experienced ≥1 bleed thereafter. Of the 11 patients remaining at Hb <100 g/L in week 4, the risk of future bleeding was just as high (3 of 11 [27%]). Hence, the risk of CRNB after week 4 was not related to the correction or persistence of anemia by week 4, suggesting that baseline anemia was only a marker for patients with a higher bleeding tendency rather than a cause of bleeding.

### 3.7 Relevance of bleeding for persistent anemia

**Figure 6:**
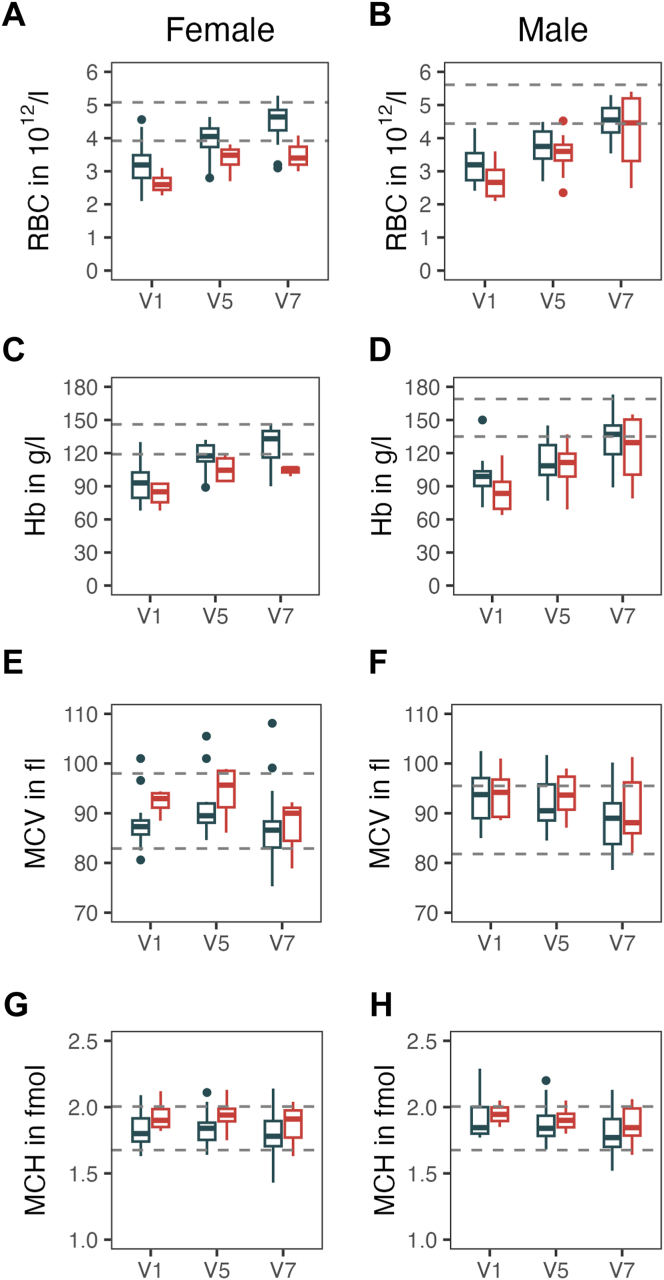
Course of anemia according to clinically relevant new bleeding during 12 weeks of emicizumab. Blue and red box plots show patients without and with clinically relevant new bleeding, respectively. (A, C, E, G) Female patients and (B, D, F, H) male patients are separated, and sex-specific reference ranges are indicated by dashed horizontal lines. Visit (V) time points are as follows: V1, baseline (day −3 to 1); V5, week 4; and V7, week 13. Hb, hemoglobin; MCH, mean corpuscular hemoglobin; MCV, mean corpuscular volume; RBC, red blood cell count.

With a median Hb of 90 g/L at baseline, most patients in the GTH-AHA-EMI study had severe anemia at baseline. Hb and RBCs remained lower throughout the study in patients with CRNB, in particular in female patients (Figure 6). Anemia was mostly normochromic normocytic, with mean corpuscular volume and MCH remaining within the normal range during the course. Markers of iron deficiency were not routinely recorded in the study.

### 3.8 Mortality and adverse events

Four (9%) of the 47 patients died during the study. This number was too low for statistical analysis of risk factors for mortality. However, we noted that patients who died were not among the oldest (70, 77, 80, and 80 years) and not among those with the most severe comorbidity (CCI, 4, 5, 6, and 7).

**Figure 7:**
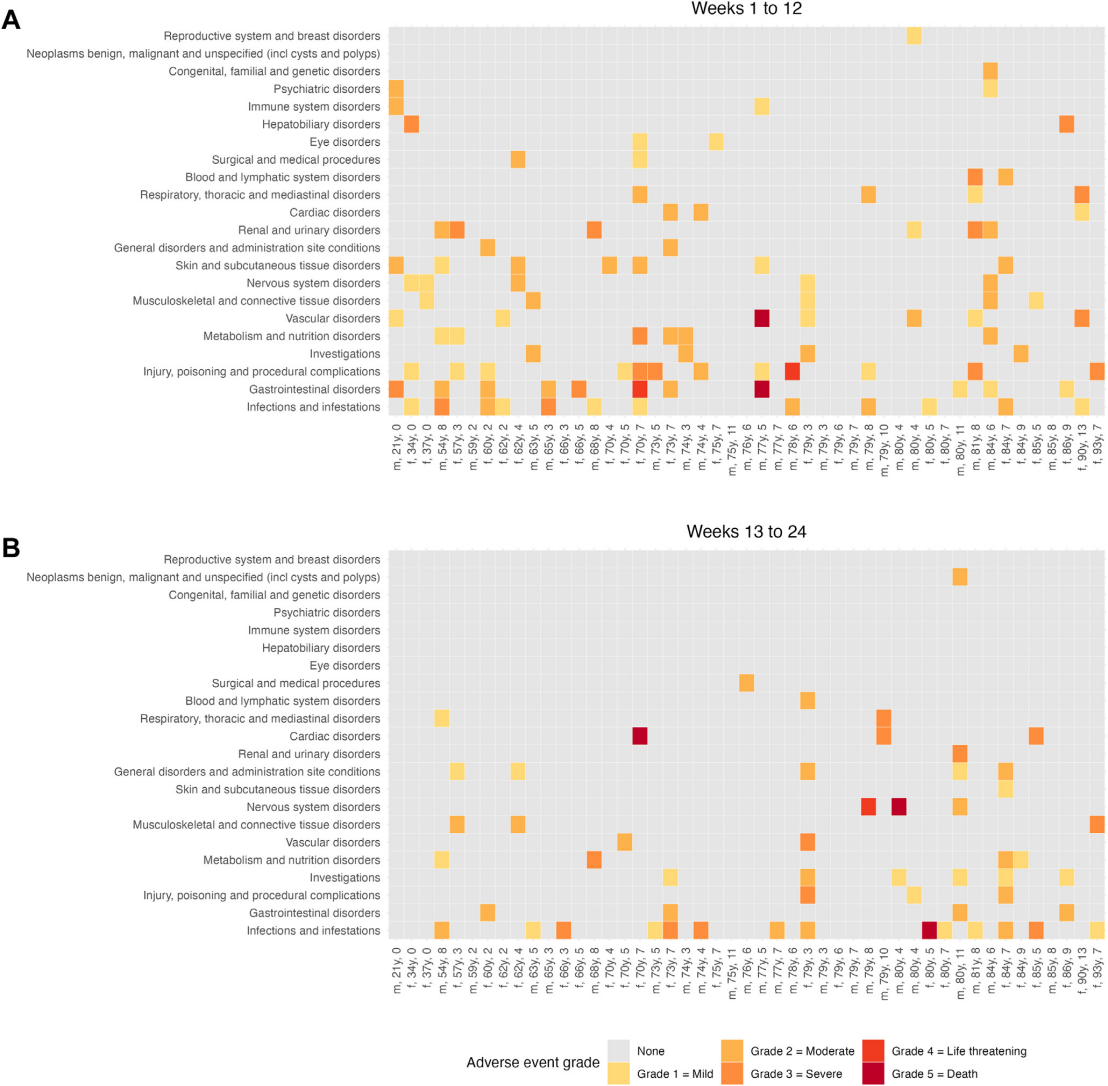
Adverse event grades according to patient age and comorbidity. Patients are sorted along the X axis according to age and denoted with their sex, age in years, and Charlson comorbidity index. Adverse event categories are sorted along the Y axis according to overall number of patients. Fill colors indicate grades of event severity. f, female; m, male.

The most frequent adverse events were infections, gastrointestinal disorders, and injuries. These events have been described in more detail in the primary publication. The overall frequency and system/organ class distribution or severity of adverse events were not related to age or CCI (Figure 7). Age and CCI were also not associated with adverse events grade ≥3 (Supplementary Table S3). Patients with adverse events grade ≥3 more often had WHO-PS ≥ 2 at baseline (14 of 16; 88%) compared with patients without such events (18 of 31; 58%; *P* = .04; Supplementary Table S3).

Sex differences in the occurrence of adverse events were only noted for the class “General disorders and administration site conditions” (6 of 23 females vs 1 of 24 males; *P* = .048). The events occurring in females were described as edema or peripheral swelling and thrombophlebitis.

We also assessed whether CRNB during 12 weeks of emicizumab was related to adverse events. Eight (57%) of 14 patients with CRNB had events of the class “Injury, poisoning, and procedural complications” compared with 7 (21%) of 33 without CRNB (*P* = .037). These events mainly included falls and injuries (7 patients) related to bleeding events. Five (36%) of 14 patients with CRNB had an event of the class “Renal and urinary disorders,” which was only seen in 2 (6%) of 33 patients without CRNB (*P* = .018). This included 2 patients with acute kidney failure in weeks 1 and 2, both related to severe anemia at baseline (65 and 78 g/L), while the other events did not appear to be causally linked to bleeding or anemia (urolithiasis, incontinence, and renal cysts).

## 4 Discussion

Prophylaxis with emicizumab has the potential to profoundly change clinical practice of AHA management [30]. The drug not only reduces the risk of bleeding but may also lessen the need for early and intense IST, thereby reducing the risk of serious adverse events and mortality [28]. However, our pivotal study also showed that emicizumab alone does not prevent all bleeds. Adverse events other than bleeding were also recorded in this elderly, fragile, and comorbid population of patients. Careful consideration of these risks will be important in managing AHA in future clinical practice.

The current study aimed to support this consideration by analyzing baseline clinical and laboratory characteristics that could be useful in identifying patients at risk of bleeding or other adverse events. The cohort of patients enrolled in this interventional clinical trial had very similar baseline characteristics to those in previous noninterventional registries. Half of our patients were >75 years old; 41% had a WHO-PS of 3 or 4. Diabetes mellitus (34%), renal disease (26%), and neoplasia (23%) were frequent. To the best of our knowledge, our approach to assessing comorbidity through planned and monitored recording of medical history items offers the most comprehensive characterization of a sizable AHA population to date.

Our analysis revealed that rates of CRNB under emicizumab were similar across subgroups of age, sex, physical performance status, and comorbidity. Baseline FVIII activity and inhibitor titer also did not appear to impact the bleeding rate while under emicizumab. In other words, emicizumab had consistent prophylactic efficacy regardless of these characteristics.

This was in sharp contrast to historical data from the GTH-AH 01/2010 study, where patients received management with IST (but no emicizumab). The bleeding risk was particularly higher in patients with poor baseline WHO-PS (3 or more) and remained high as long as FVIII activity was reduced [31]. The availability of effective bleed prophylaxis, even for patients with severe comorbidity, poor performance status, and very low FVIII, is a true breakthrough in the management of AHA.

The risk of CRNB during prophylaxis with emicizumab appeared to be somehow linked to anemia at baseline. Evidence for this notion was significantly lower Hb, RBCs, and MCH at baseline in those patients who later had CRNB during the efficacy period of 12 weeks. Anemia has not only been described as a consequence of bleeding but also as a risk factor [32]. Experimental data suggest that platelet adhesion increases 5-fold as hematocrit values increase from 10% to 40% [33]. Patients with hematological malignancy, renal insufficiency, and inherited or acquired disorders of primary hemostasis are at risk of anemia-induced bleeding [34]. Anemia-induced bleeding has also been described in the context of primary postpartum hemorrhage [35]. Even healthy donors show a 15% prolongation of bleeding time after apheresis of 2 units of RBCs [36].

We were therefore interested in examining the longitudinal course of anemia and bleeding events in our patient cohort more closely. The results were less clear, as the proportion of patients bleeding after week 4 was similar, regardless of whether their Hb had improved at week 4. This may imply that anemia is not a risk factor for bleeding per se in these patients but rather a consequence of a more severe bleeding tendency upfront. However, caution is required due to the low number of patients with bleeding after week 4 and the lack of further Hb data during the interval between week 4 and the actual bleeding. Anemia persisted longer and more severely in patients with CRNB. This finding is important because anemia can cause fatigue but also organ damage, with the kidney being particularly sensitive [37]. The 2 patients with acute renal failure in our study had severe anemia. We suggest that anemia as a risk factor for bleeding and other adverse events is addressed more directly in future studies.

We also observed that several events of breakthrough bleeding were related to injury from falls. This should be actively addressed with patients, medical professionals, and caregivers because awareness of the risk of bleeding from injury can help to prevent it.

Medication with ASA or other antithrombotic drugs was stopped in most cases around the time of study enrollment. We identified 3 cases where ASA may have potentially provoked bleeding, always during times when emicizumab levels were presumably very low. Other case reports should be acknowledged, suggesting that persons with AHA needing cardiovascular interventions have been medicated safely with ASA while under prophylaxis with emicizumab [21,22].

The risk of adverse events other than bleeds is also considerable in persons with AHA. We found that the risk of severe adverse events (grade ≥3) was linked to a poor baseline WHO-PS. This was consistent with data from our previous study, where patients with poor baseline WHO-PS also had an increased risk of adverse events and mortality [6].

Our study has limitations that should be discussed. First, the number of patients with CRNB (14 out of 47) and the rate of bleeding (0.04 events per patient-week) may have been too small to detect risks associated with single comorbidities or other baseline characteristics. Some associations that were noted might have been chance findings because of multiple testing and the risk of statistical type 1 error. In particular, the observed interaction of anemia and the risk of bleeding needs further study.

In conclusion, our study reports consistently low bleeding rates under emicizumab prophylaxis, even in patients of very advanced age, frailty, and heavy comorbidity. However, a small risk of even fatal breakthrough bleeding exists, and this cannot be predicted by clinical or laboratory baseline characteristics. IST will continue to play a significant role in the management of AHA, but its timing and intensity should be reconsidered in light of the new opportunities presented by emicizumab.
