# Supplementary material for: Comorbidity and adverse events in acquired hemophilia A: data from the GTH-AHA-EMI study
Source: Res Pract Thromb Haemost. 2024 Sep 5;8(7):102565. doi: 10.1016/j.rpth.2024.102565 (PMC11480235; doi:10.1016/j.rpth.2024.102565)
Supplement: Supplementary material [file mmc1.pdf]

Supplement information

Comorbidity and adverse events in acquired hemophilia A: data from the GTH-AHA-EMI study

Authors and affiliations

Christian Herbert Burgmann,[1] Ulrich J. Sachs,[2] Karolin Trautmann-Grill,[3] Christian Pfrepper,[4] Paul Knöbl,[5] Richard Greil,[6] Johannes Oldenburg,[7] Wolfgang Miesbach,[8] Katharina Holstein,[9] Hermann Eichler,[10] Patrick Möhnle,[11] Matthias Höpting,[12] Christiane Dobbelstein,[1] Robert Klamroth,[13] and Andreas Tiede[1]

1. Hematology, Hemostasis, Oncology, and Stem Cell Transplantation, Hannover Medical School, Hannover, Germany
2. Institute for Clinical Immunology and Transfusion Medicine, Justus Liebig University, Giessen, Germany
3. Medical Clinic I, University Hospital Carl Gustav Carus, TU Dresden, Dresden, Gemany
4. Division of Hemostaseology, Medical Department I, University Hospital Leipzig, Leipzig, Germany
5. Department of Medicine 1, Division of Hematology and Hemostasis, Medical University of Vienna, Vienna, Austria
6. IIIrd Medical Department, Paracelsus Medical University Salzburg, Salzburg Cancer Research Institute-CCCIT; Cancer Cluster Salzburg, Salzburg, Austria
7. Institute of Experimental Hematology and Transfusion Medicine, University Clinic Bonn, Bonn, Germany
8. Medical Clinic II, Institute of Transfusion Medicine, Goethe University, Frankfurt, Germany
9. Hematology and Oncology, University Medical Center Hamburg-Eppendorf, Hamburg, Germany
10. Institute of Clinical Hemostaseology and Transfusion Medicine, Saarland University and University Hospital, Homburg/Saar, Germany
11. Department of Transfusion Medicine, Cellular Therapeutics and Hemostaseology, Department of Anesthesiology, Hospital of Ludwig Maximilian University, Munich, Germany
12. Department of Hematology and Oncology, University Hospital Regensburg, Regensburg, Germany
13. Internal Medicine, Vivantes Clinic Friedrichshain, Berlin, Germany

Correspondence and requests for data sharing

Andreas Tiede (MD, PhD), Hannover Medical School, Carl Neuberg Str. 1, 30625 Hannover, Germany

Tel.: + 49-511-532-4147, fax: + 49-511-532-18524, email: tiede.andreas@mh-hannover.de

Supplement Table S1

Association between clinical baseline characteristics and CRNB during study.

Characteristic	Patients with CRNB N = 14[1]	Patients without CRNB N = 33[1]	P value[2]
Age			0.587
>75 years	8 (57%)	16 (48%)	
≤75 years	6 (43%)	17 (52%)	
Sex			0.069
female	4 (29%)	19 (58%)	
male	10 (71%)	14 (42%)	
Performance status			>0.999
WHO 0-1	4 (29%)	11 (33%)	
WHO 2-4	10 (71%)	22 (67%)	
Comorbidity			0.966
CCI 0-4	5 (36%)	12 (36%)	
CCI 5-13	9 (64%)	21 (64%)	
Body mass index			0.665
<18.5 kg/m²	2 (14%)	3 (9.1%)	
18.5-24.9 kg/m²	7 (50%)	12 (36%)	
25-29.9 kg/m²	4 (29%)	12 (36%)	
≥30 kg/m²	1 (7.1%)	6 (18%)	
Baseline hemoglobin			0.077
<100 g/l	13 (93%)	22 (67%)	
≥100 g/l	1 (7.1%)	11 (33%)	

[1]n (%)

[2]Pearson's Chi-squared test; Fisher's exact test

Abbreviations: CRNB, clinically relevant new bleeds

Supplement Table S2

Baseline laboratory values in patients with and without CRBN during 12 weeks after starting emicizumab

Analyte	Patients with bleeds N = 14[1]	Patients without bleeds N = 33[1]	P value[2]
Factor VIII (IU/dl)	3 (0, 5)	1 (0, 6)	0.413
Inhibitor (BU/ml)	6 (2, 29)	17 (5, 59)	0.218
Hemoglobin (g/l)	84 (70, 94)	97 (81, 104)	**0.030**
Red blood cells (10^{12}/l)	2.65 (2.30, 3.04)	3.19 (2.80, 3.54)	**0.008**
MCH (fmol)	1.93 (1.89, 2.00)	1.82 (1.77, 1.94)	**0.020**
MCV (fl)	93.1 (89.3, 96.1)	88.7 (87.0, 96.2)	0.071
MCHC (mmol/l)	20.70 (20.12, 21.27)	20.48 (20.11, 21.04)	0.449
Leukocytes (10^{9}/l)	10.3 (9.2, 12.6)	10.2 (8.2, 12.2)	0.753
Platelets (10^{9}/l)	305 (230, 400)	315 (246, 406)	0.972

[1]Median (IQR)

[2]Wilcoxon rank sum test

Abbreviations: CRNB, clinically relevant new bleeds; MCH, mean corpuscular hemoglobin; MCV, mean corpuscular volume; MCHC, mean corpuscular hemoglobin concentration

Supplement Table S3

Association between clinical baseline characteristics and the occurrence of adverse events of grade 3 or more (bleeding events excluded).

Characteristic	Adverse events grade 3 or more		P value[2]
	**No,** N = 31[1]	**Yes,** N = 16[1]	
Age			0.471
>75 years	17 (55%)	7 (44%)	
≤75 years	14 (45%)	9 (56%)	
Sex			0.471
female	14 (45%)	9 (56%)	
male	17 (55%)	7 (44%)	
Performance status			0.040
WHO 0-1	13 (42%)	2 (13%)	
WHO 2-4	18 (58%)	14 (88%)	
Comorbidity			0.252
CCI 0-4	13 (42%)	4 (25%)	
CCI 5-13	18 (58%)	12 (75%)	
Body mass index			0.190
<18.5 kg/m²	2 (6.5%)	3 (19%)	
18.5-24.9 kg/m²	13 (42%)	6 (38%)	
25-29.9 kg/m²	13 (42%)	3 (19%)	
≥30 kg/m²	3 (9.7%)	4 (25%)	
Hemoglobin			0.176
<100 g/l	21 (68%)	14 (88%)	
≥100 g/l	10 (32%)	2 (13%)	

[1]n (%)

[2]Pearson's Chi-squared test; Fisher's exact test
